# Supplementary material for: Variant surface glycoprotein density defines an immune evasion threshold for African trypanosomes undergoing antigenic variation
Source: Nat Commun. 2017 Oct 10;8:828. doi: 10.1038/s41467-017-00959-w (PMC5635023; doi:10.1038/s41467-017-00959-w)
Supplement: Supplementary file 1 — Supplementary Information [file 41467_2017_959_MOESM1_ESM.pdf]

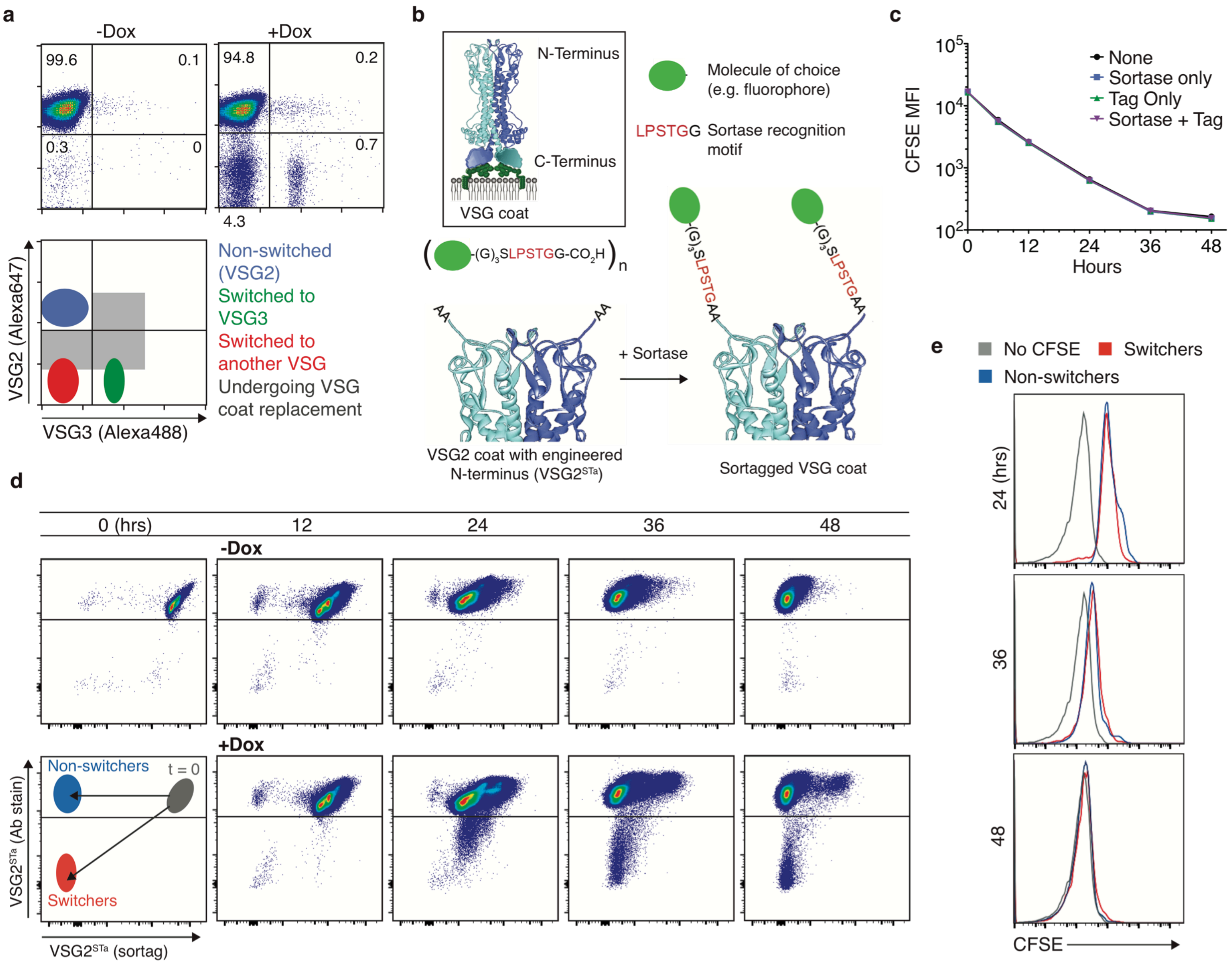

Supplementary Figure 1: Determining the rate of VSG loss in switching and non-switching trypanosomes. a, VSG switching in induced (Dox+) and non-induced (Dox-) Δ70 cells at 48hrs. **b**, Illustration of VSG sortagging. **c**, Lack of effect of sortagging on cell growth rate. Data show flow cytometry measurements of population mean fluorescence intensity (MFI) of CFSE. CFSE is an intracellular fluorescent stain that dilutes as a result of cell proliferation, and is commonly used as a proliferation metric[1]. Experimental groups were treated with HMI-9 alone ("None"), or HMI-9 with sortase enzyme, fluorescent tag, or both sortase and tag, then with CFSE (see methods) then incubated in normal culture conditions. Samples were then isolated from these cultures at regular inteverals for analysis. **d**, $VSG2^{STa}$ loss over time in sortagged and induced (+Dox) or non-induced (-Dox) $\Delta70^{STa}$ cells. Line shows the gate between "Switcher" and "Non-switcher" populations. **e**, Relative growth rates of Switcher and Non-switcher populations measured by CFSE staining. The shoulder of slightly slower growing cells in the non-switching population (most visible at 24 hrs) likely accounts for the slightly increased $t_{E1/2}$ in +Dox non-switchers, and may be the result of a cell cycle arrest in cells repairing the induced DNA double strand break[2]. A corresponding population with slow sortag loss can be seen in **d**. Data are representative of at least three (**a** and **d**), two (**e**), or one (**c**) replications of these experiments. VSG image in **b** was adapted from a previous publication[3] with permission from the author.

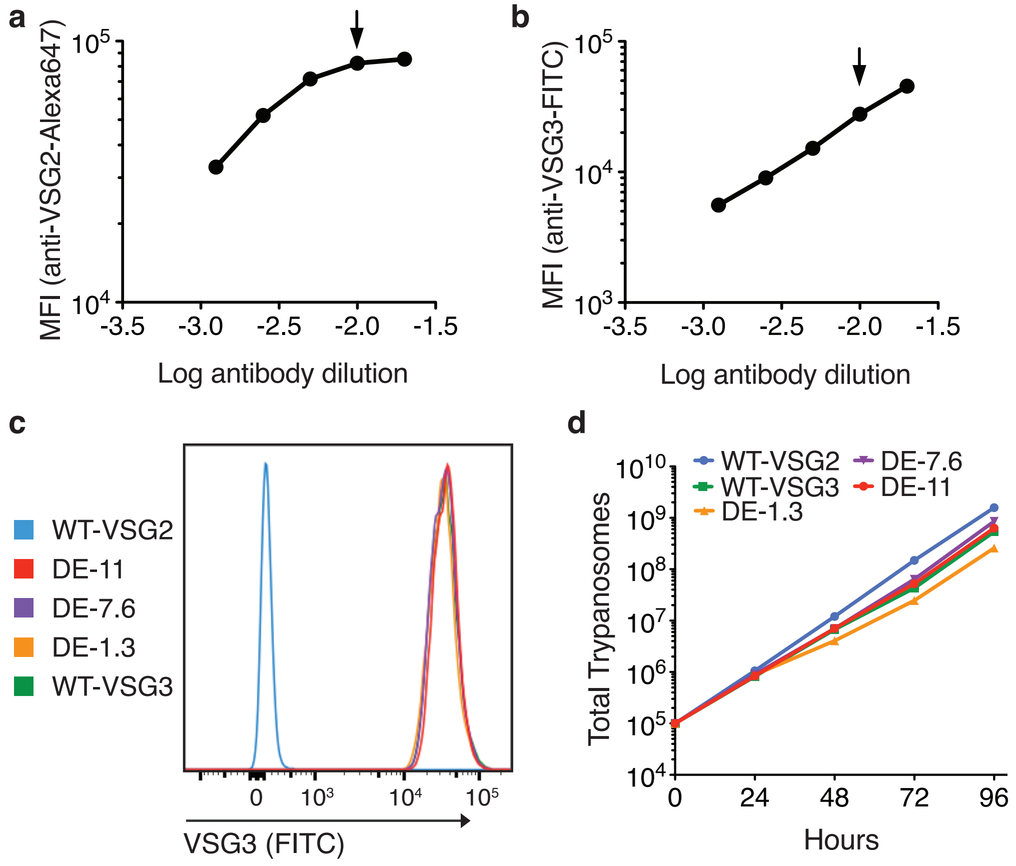

Supplementary Figure 2: Flow cytometry-based measurements of VSG surface expression. **a**, Titration curve of monoclonal anti-VSG2-Alexa647 Ab staining of WT-VSG2 cells. Data are represented as population mean fluorescence intensity (MFI) measured via flow cytometry (see methods for sample preparation). Saturating levels of VSG2 staining were reached with this Ab; arrow indicates the concentration used for calculation of relative surface VSG2 expression in DE clones (Fig. 2b). **b**, Titration curve (as in **a**) of monoclonal anti-VSG3-FITC Ab staining of WT-VSG3 cells. After several fluorophore conjugation and Ab concentration attempts, we were unable to obtain a saturating concentration of this Ab. However, analysis of anti-VSG3-FITC staining at non-saturating levels, demonstrated in **c** (at concentration indicated by arrow in **b**), indicated that the amounts of surface VSG3 expressed by WT-VSG3 cells and each of the DE clones are approximately equal. Unlike the VSG2 ratio measurements (Fig. 2B), this result should be valid even without saturating conditions, as cells with equal VSG3 staining will not differentially affect the free anti-VSG3 Ab concentration present during staining. **d**, Growth rate analysis of WT-VSG2, WT-VSG3, and DE clones. The experiment shown in **c** was performed once, and results are representative of another experiment using the same Ab at an 8-fold lower concentration, and experiments with the same monoclonal Ab conjugated to two additional fluorophores (Alexa488 and Pacific Blue; at least one experiment at one dilution of each conjugate, titrated within the linear range). Experiments shown in **a**, **b**, and **d** were performed once.

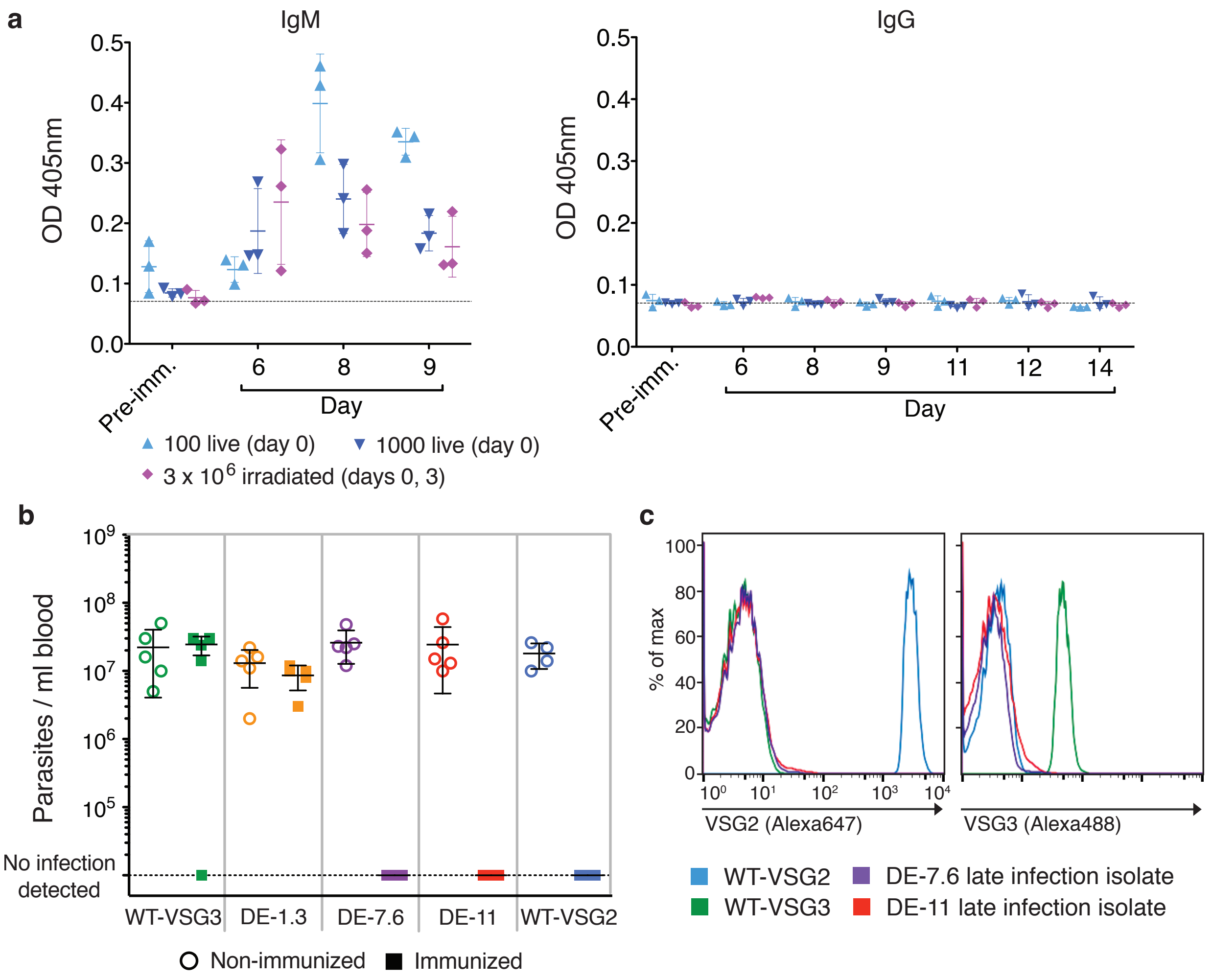

Supplementary Figure 3: Immunization-challenge experiments with DE clones reveal a threshold of immune recognition. a, VSG2-specific IgM and IgG titers elicited by live or UV irradiated trypanosomes following single or dual immunizations as indicated. Data show relative titers before (Pre-imm.) and after immunization as measured by ELISA (mean ± SD, n = 3 mice/group). Dotted lines indicate average background OD in wells incubated with no serum. This experiment was performed once. **b**, Levels of parasitemia at day 5 post-challenge in immunized and non-immunized mice (mean ± SD, n = 5 mice/group). Immunized and non-immunized parasitemia levels are not significantly different ($P > 0.5$) in mice showing parasitemia with WT-224 and DE-1.3 clones, as determined by *t*-test (variances are similar between groups, as determined by *F*-test). These data were collected in one experiment. **c**, Lack of VSG2 and VSG3 expression in late-arising (beyond day 8) DE-7.6 and DE-11 infections in pre-immunized mice. Data show flow cytometry analysis of trypanosomes isolated from the blood of infected mice on day 15 post-challenge, along with control VSG2 and VSG3-expressing trypanosomes (WT-VSG2 and WT-VSG3, respectively). The data shown are representative of late-infection trypanosome isolates from all DE-7.6 and DE-11 infections observed in pre-immunized mice (1 infection in DE-7.6 mice, 6 infections in DE-11 mice; one mouse from each group did not present parasitemia high enough for trypanosome isolation and analysis).

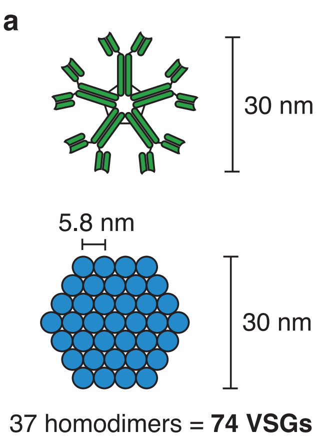

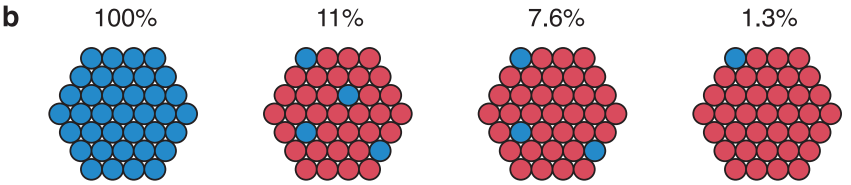

% initial VSG remaining	# of initial VSGs within reach of a single IgM	
100.0	74.0	Multiple IgM-VSG contacts likely
11.0	8.1	
7.6	5.6	
1.3	1.0	Multiple IgM-VSG contacts unlikely

Supplementary Figure 4: The effect of VSG density on IgM binding ability. (**a** and **b**) Identical to main text Fig. 4B and C, except the model is presented using an IgM diameter of 30nm instead of 40nm.

VSG effective half-life ($t_{E1/2}$)	$\frac{1}{t_{E1/2}} = \frac{1}{t_D} + \frac{1}{t_{1/2}}$
Initial VSG remaining over time during coat replacement	$N = \frac{N_0}{2^{(t / t_{E1/2})}} = \frac{N_0}{2^{(t / t_D) + (t / t_{1/2})}}$

Supplementary Table 1: VSG turnover equations.

VSG effective half-life: $t_{E1/2}$ = VSG effective half-life. t_D = trypanosome population doubling time. $t_{1/2}$ = VSG protein half life. Both dilution of VSGs through cell division and VSG loss through shedding and degradation contribute to the total rate of VSG loss from each individual trypanosome[4]. The distinct contributions of each of these processes are defined by t_D and $t_{1/2}$, respectively. The combined half-life value ($t_{E1/2}$) is mathematically equivalent to the sum of the inverses of these partial half-life values. The t_D of our $\Delta 70^{STa}$ clone is 7.2 hrs (data not shown). Using this doubling time and the previously published $t_{1/2}$ of 32 hrs[4,5], our estimated expected $t_{E1/2}$ value is ~5.9 hrs.

Initial VSG remaining over time during coat replacement: N_0 = total initial VSG at time t = 0 (e.g. ~10^7 VSG monomers, 5 x 10^6 VSG dimers, or 100%). t = time since genetic VSG switch occurred. N = amount or percent of initial VSG remaining after time t. By definition, the amount of initial VSG remaining on each trypanosome (N) reduces by half after each time interval equal to $t_{E1/2}$. The equation shown here thus defines the initial VSG remaining at each time t following the genetic VSG switch. For example, if $t = t_{E1/2}$, the formula shown returns a value of initial VSG remaining $N = (1/2)N_0$, and if $t = (2)t_{E1/2}$, the formula returns a value of $N = (1/4)N_0$. The formula shown on the right is produced by combining the first formula with the prior equation relating $t_{E1/2}$ to t_D and $t_{1/2}$.

Using the values $N_0 = 10^7$ VSG monomers (a full VSG coat prior to genetic VSG switch), and $t_{E1/2}$ = 4.6 hr (the average of our calculated values), the estimated time to reach N = 1 VSG remaining is 107 hrs (~4.5 days). Beyond this point (N = 1 VSG), trypanosomes with fully switched coats will arise. If N_0 = 100%, the estimated times to reach N = 7.6% and N = 1.3% are 17.1 and 28.8 hrs, respectively.

These equations were adapted[4] for ease of interpretation in the context of this study.

Supplementary Methods:

Sortase A purification

Plasmid containing the *S. pyogenes*-derived sortase A expression construct (pSpSortA-pET28a[6], a gift of Dr. Assaf Raz, Rockefeller University) was transformed into BL21 DE3 cells (Life Technologies C6000-03). Colonies from this transformation were used to inoculate large cultures of LB media (VWR 1.10285.0500) and grown shaking at 37°C to an optical density (OD_{600}) of 0.4 - 0.8. Cultures were then induced with 1mm IPTG, grown an additional 3-4hr, and harvested by centrifugation. Cell pellets were resuspended in TBS/imidazole (20mM Tris, 150mM NaCl, 20mM imidazole), and lysed using an EmulsiFlex-C5 homogenizer (Avestin). DNAse-A powder (Sigma D5025) and 5mM BME were added to the lysate, and it was centrifuged to remove particulate. The supernatant was poured over a column of Ni chelating beads (Qiagen D-40724) which had been equilibrated with wash buffer (20mM Tris, 300mM Nacl, 20mM imidazole, 5mM BME). The column was washed with 100ml wash buffer and eluted with 30-35ml elution buffer (20mM Tris, 300mM NaCl, 200mM imidazole, 5mM BME). Samples containing protein were pooled and dialyzed in (20mM Tris, 150mM NaCl, 1mM DTT). The resulting sample was concentrated using a centrifugal filter unit (Amicon Ultra – 10,000 NMWL, Millipore), aliquoted, and frozen for future use.

Generation of DE clones (Fig. 2a)

1) Clone DE-1.3 (plasmid pJP04):

The modified procyclin (Procyclin (mod)) 5'UTR was based on the pNS10/54 5'UTR published by Siegel et al., 2005[7]. It was created by assembly PCR using overlapping oligos, followed by insertion into a pGEM backbone. The full Procyclin (mod) sequence is shown below. This 5'UTR was amplified with primers JPC18_GibsonP2For and JPC19_GibsonP2Rev. The VSG2 sequence was amplified from pSY37*[8] with primers JP09_VSG221_For and JPC24_GibsonP3221UTR_Rev. The BSD cassette (*BSD* gene with surrounding actin 5' and 3'UTRs) was amplified from pHJ37-BSD (derivative of pHJ37*, unpublished) with primers JP11_ACT5UTRStart_For and JPC22_GibsonP5Rev.

2) Clone DE-7.6 (plasmid pJP05):

VSG2 with its 5' and 3' UTRs was amplified from pSY37* with primers JPC26_GibsonP3221UTR_For and JPC24_GibsonP3221UTR_Rev. The BSD cassette was amplified as in clone DE-1.3.

3) Clone DE-7.6 (plasmid pJP07):

The Aldolase 5'UTR was amplified from pSY37* with primers JPC31_JP07P2_For and JPC32_JP07P2_Rev. VSG2 was also amplified from pSY37 with primers JP09_VSG221_For and JPC20_GibsonP3Rev. The Aldolase 3'UTR was amplified from pLEW100*[9] with primers JP10_Ald3UTR_For and JPC21_GibsonP4Rev. The BSD cassete was amplified as in clones DE-1.3 and DE-7.6.

*These plasmids were provided by Dr. Hee-sook Kim, Rockefeller University

Growth rate analysis (Supplementary Fig. 2d)

WT-VSG2, WT-VSG3, and DE clone trypanosomes were diluted to 10^5 cells/ml in culture. Cell concentration was analyzed every 24 hours, and cultures were diluted back to 10^5 cells/ml immediately after measuring concentration. Total cell number was calculated by multiplying the concentration each day by the dilution factors of each previous day.

Analysis of VSG expression in infection isolates (Supplementary Fig. 3c)

Trypanosomes were isolated from mouse blood during some infections for flow cytometry-based VSG expression analysis. For these analyses, 100 ul infected mouse blood was collected and incubated twice with 1 ml Red Blood Cell Lysing Buffer (Sigma R7757) for 4 minutes at room temperature. Samples were then resuspended in 100 ul HMI-9 with a 1:50 dilution of Mouse FC Block (BD 553142) and incubated on an inversion rotator for 10 minutes at 4°C (all subsequent steps were on ice or at 4°C). 10 ul of each sample was then added to 100 ul of HMI-9 containing anti-VSG2-Alexa647 (1:1000 dilution) and anti-VSG3-Alexa488 (1:200 dilution) antibodies. These solutions were incubated on an inversion rotator for 10 minutes, pelleted, and washed once with 1 ml HMI-9. Samples were then pelleted again, resuspended in 200 ul HMI-9, and analyzed using a BD-FACSCalibur flow cytometer. Control WT-VSG2 and WT-VSG3 samples were pelleted from culture and stained identically with the same anti-VSG2 and anti-VSG3 antibody dilutions.

Primers (Sequences 5’->3’)

JPC1_UniqueFor: TATAAGATCTGCGGCCGCGAGTAAGTAAGTTGGAGCGCAC
JPC2_UniqueRev: ATATCTCGAGGCGGCCGCCATGCTGTCGGTTGTCGGACTTGG
JPC3_pUC19For: TATACTCGAGCCGGGAGCTGCATGTGTCAGAGG
JPC4_pUC19Rev: ATATAGATCTTCGTTCGGCTGCGGCGAGCGG
JPC18_GibsonP2For:
GGGAGGACTAACACAATGAATACGGTTTACACCGGTGGGCTGCACGCGCCTTCGAG
TTTT
JPC19_GibsonP2Rev:
TGACATTACCATTCCGGTACTGTTGGTAAAATGCCTTCCAATCAGGAGGCCCGGCTT
TTC
JP09_VSG221_For : ATGCCTTCCAATCAGGAGGCC
JPC24_GibsonP3221UTR_Rev:
GACTGTCTGATTGTCTAGAAATATTTCTGGCAGGGCACAGCAAGGTCTTCTGAAAT
TCATGT
JP11_ACT5UTRStart_For: GGGCACAGCAAGGTCTTCTGA
JPC22_GibsonP5Rev:
CGTTTGTTATCTATGCAGTATTCTGCAGGCGTGATGAAGATGCAGATAGATGGCATA
GAT
JPC26_GibsonP3221UTR_For:
GGGAGGACTAACACAATGAATACGGTTTACATTGCATTTGGACACCGCTATACATAT
GTT
JPC31_JP07P2_For:
GGGAGGACTAACACAATGAATACGGTTTACGTGCTCAAGCTGTGTAGCGCACGCGT
TTCC
JPC32_JP07P2_Rev:
AGCCCGAAAACATAAACTCAACTGCAACGAATGCCTTCCAATCAGGAGGCCCGGCT
TTTC
JPC20_GibsonP3Rev:
CCTCTTTGGCTTGCAGTTTTGCTTTTTTAAGATCCTGCCCATTTAGTTGGCTTTTCCCT
T
JP10_Ald3UTR_For: GATCCTGCCCATTTAGTTGGC
JPC21_GibsonP4Rev:
GCCACTACAAGTTGGTTTCCTTCCCCTGCAGGGCACAGCAAGGTCTTCTGAAATTCA
TGT

Procyclin UTR (mod) sequence

ACCGGTGGGCTGCACGCGCCTTCGAGTTTTTTTCCTTTTCCCCATTTTTTTCAACTTG
AAGGGTACCCTTCAATTACACCAAAAAATAAAATTCACAAACTTGGAATTCCTTTGT
GTTACATTCTTGATCGCTCGCACTGACATTACCATTCCGGTACTGTTGGTAAA

References:

1. Lyons, A. B. & Parish, C. R. Determination of lymphocyte division by flow cytometry. *Journal of Immunological Methods* **171,** 131–137 (1994).
2. Glover, L., Alsford, S. & Horn, D. DNA Break Site at Fragile Subtelomeres Determines Probability and Mechanism of Antigenic Variation in African Trypanosomes. *PLoS Pathog* **9,** e1003260 (2013).
3. Engstler, M. *et al.* Hydrodynamic Flow-Mediated Protein Sorting on the Cell Surface of Trypanosomes. *Cell* **131,** 505–515 (2007).
4. Seyfang, A., Mecke, D. & Duszenko, M. Degradation, recycling, and shedding of Trypanosoma brucei variant surface glycoprotein. *J. Protozool.* **37,** 546–552 (1990).
5. Bülow, R., Nonnengässer, C. & Overath, P. Release of the variant surface glycoprotein during differentiation of bloodstream to procyclic forms of Trypanosoma brucei. *Mol. Biochem. Parasitol.* **32,** 85–92 (1989).
6. Raz, A. & Fischetti, V. A. Sortase A localizes to distinct foci on the Streptococcus pyogenes membrane. *Proc. Natl. Acad. Sci. U.S.A.* **105,** 18549–18554 (2008).
7. Siegel, T. N., Tan, K. S. W. & Cross, G. A. M. Systematic Study of Sequence Motifs for RNA trans Splicing in Trypanosoma brucei. *Molecular and Cellular Biology* **25,** 9586–9594 (2005).
8. Schulz, D., Zaringhalam, M., Papavasiliou, F. N. & Kim, H.-S. Base J and H3.V Regulate Transcriptional Termination in Trypanosoma brucei. *PLoS Genet* **12,** e1005762–21 (2016).
9. Wirtz, E., Leal, S., Ochatt, C. & Cross, G. A. A tightly regulated inducible expression system for conditional gene knock-outs and dominant-negative genetics in Trypanosoma brucei. *Mol. Biochem. Parasitol.* **99,** 89–101 (1999).
